# An early-detected intraductal papillary squamous neoplasm of the pancreas under peroral pancreatoscopy

**DOI:** 10.1055/a-2587-8777

**Published:** 2025-05-28

**Authors:** Shanshan Shen, Shuang Nie, Lei Wang

**Affiliations:** 1 66506 Department of Gastroenterology, Nanjing Drum Tower Hospital, Affiliated Hospital of Medical School, Nanjing University, Nanjing, Jiangsu, China

A 61-year-old woman presented with dull upper abdominal pain, notably postprandial. Routine upper gastrointestinal endoscopy and laboratory tests were unremarkable. Enhanced computer tomography (CT) scan and magnetic resonance imaging (MRI) simply revealed mild dilation of the pancreatic duct. Endoscopic ultrasound (EUS), however, identified a 5 mm hypoechoic mass in the head of the pancreatic duct, might contribute to the ductal dilation ( Fig. 1 ). Further evaluation with endoscopic retrograde pancreatography (ERP) and peroral pancreatoscope (POP) confirmed a mass with a red surface and vascular proliferation in the head of the pancreatic duct, occupying two-thirds of the lumen, with the remaining pancreatic duct appearing normal ( Fig. 2 , Video 1 ). Targeted biopsy under direct visualization revealed extensive squamous metaplasia.

**Fig. 1:**
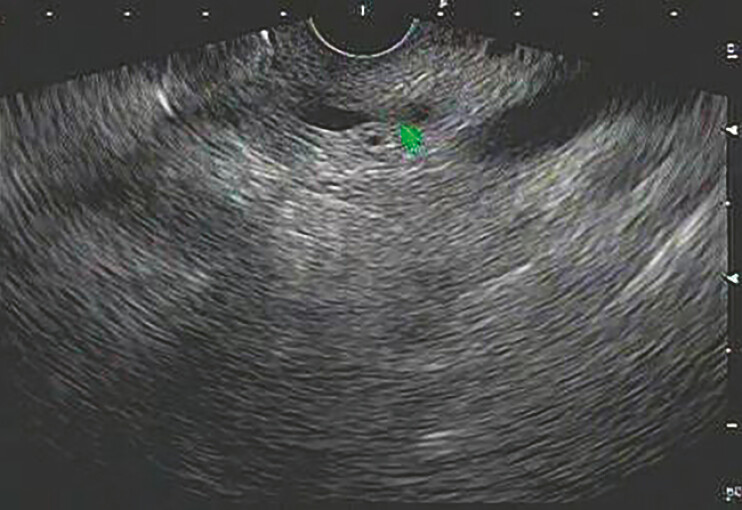
EUS photography showed pancreatic duct’s dilation, with a suspected hypoechoic mass measuring 5 mm in the head of the pancreatic duct.

**Fig. 2:**
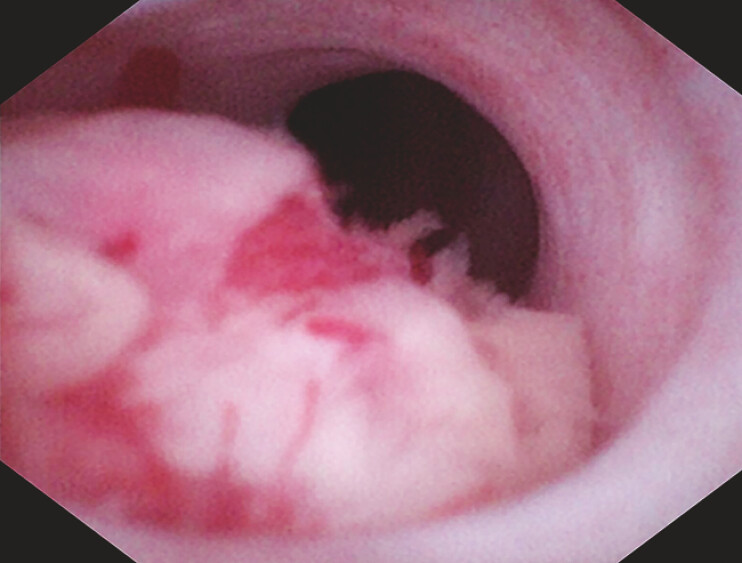
POP photography showed the presence of a mass in the head of the pancreas, with red surface and vascular proliferation, occupying 2/3 of the lumen.

An early-detected intraductal papillary squamous neoplasm of the pancreas under peroral pancreatoscopy.Video 1

Given the abnormal signs under POP, a pre-malignant lesion was suspected. Therefore, the patient underwent the laparoscopic duodenum-preserving pancreatic head resection, pancreaticojejunostomy, and abdominal lymphadenectomy ( Fig. 3 ). Histopathology of the resection specimen showed a solid papilloma with squamous metaplasia, with localized highly atypical hyperplasia, confined to the ductal epithelium without parenchyma involvement ( Fig. 4 ). Immunohistochemical staining showed the positive result of P40 in the lesional cells ( Fig. 5 ), indicating squamous differentiation, a rare pathology in pancreatic intraductal tumors 1 .

**Fig. 3:**
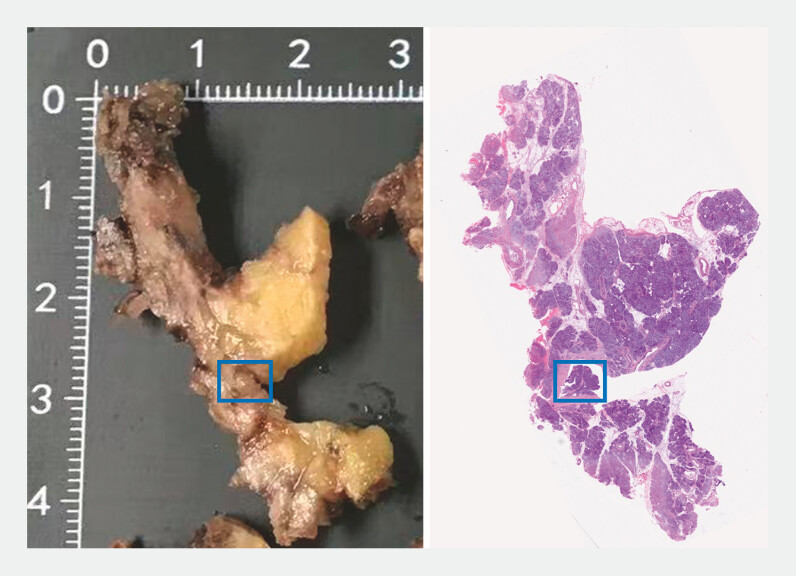
Laparoscopic appearance and histopathologic view of the intact specimen and the tagged tumor.

**Fig. 4:**
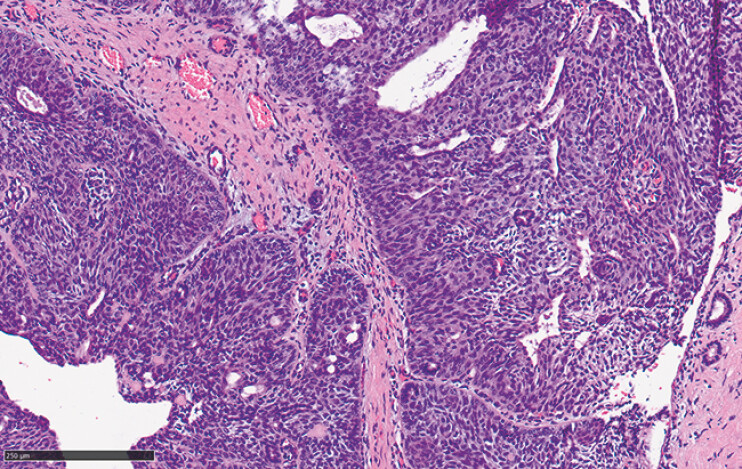
HE staining showed solid papilloma, with local squamous metaplasia of ductal epithelium, and the local squamous epithelium was highly atypical hyperplasia. Scale bar: 250 µm.

**Fig. 5:**
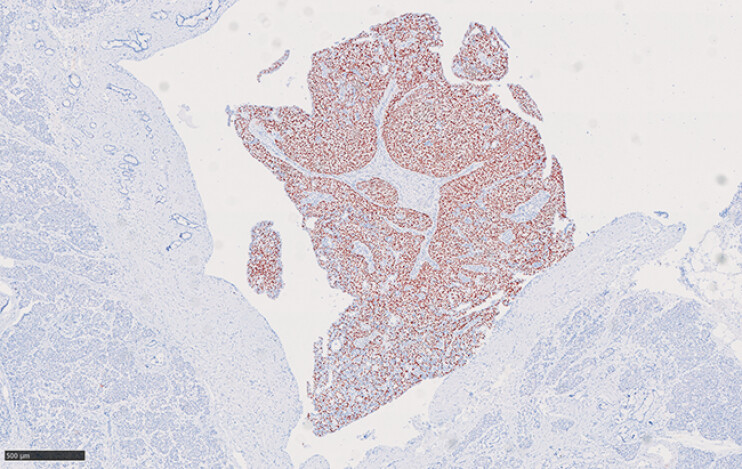
Immunohistochemical staining showed that tumor cells expressed P40. Scale bar: 500 µm.

This case represents the smallest pancreatic intraductal tumor discovered through EUS and POP ever reported, especially the rare pathology of squamous neoplasm, marking the first documented case worldwide. The patient had an uneventful recovery and remained asymptomatic at the 3-month follow-up.

We highlight this case to underscore the pivotal role of EUS combined with POP in the early detection and characterization of pancreatic intraductal tumors, enabling timely radical intervention, improving patient outcomes, and expanding our understanding of rare pancreatic pathologies.

Endoscopy_UCTN_Code_CCL_1AZ_2AB

## Notes

Endoscopy E-Videos https://eref.thieme.de/e-videos
*E-Videos* is an open access online section of the journal *Endoscopy* , reporting on interesting cases and new techniques in gastroenterological endoscopy. All papers include a high-quality video and are published with a Creative Commons CC-BY license. Endoscopy E-Videos qualify for HINARI discounts and waivers and eligibility is automatically checked during the submission process. We grant 100% waivers to articles whose corresponding authors are based in Group A countries and 50% waivers to those who are based in Group B countries as classified by Research4Life (see: https://www.research4life.org/access/eligibility/ ). This section has its own submission website at https://mc.manuscriptcentral.com/e-videos .
